# Development and psychometric evaluation of the Brief Parenting Questionnaire

**DOI:** 10.1186/s40359-025-02395-8

**Published:** 2025-01-29

**Authors:** Kenneth E. Miller, Alexandra Chen, Ioannis Bakolis, Gabriela V. Koppenol-Gonzalez, Maguy Arnous, Fadila Tossyeh, Dalia Al-Ogaily, Mark J. D. Jordans

**Affiliations:** 1 Department of Educational and Counselling Psychology and Special Education, The University of British Columbia, Vancouver, BC 2125 Main Mall Canada; 2 Department of Psychology, Harvard University, Cambridge, MA USA; 3 Health Service and Population Research Department, Institute of Psychiatry, Psychology and Neuroscience, King’s College London, London, UK; 4 Department of Research and Development, War Child Alliance, Amsterdam, The Netherlands; 5 War Child Alliance, Lebanon Offices, Beirut and Tripoli, Lebanon; 6 Amsterdam Institute of Social Science Research, University of Amsterdam, Amsterdam, The Netherlands

**Keywords:** Parenting, Measurement, Stress, Armed conflict, LMIC, Refugees, Child mental health

## Abstract

**Background:**

There is a paucity of brief self-report parenting measures validated for use in low- and middle-income countries (LMICs). We developed the Brief Parenting Questionnaire (BPQ), a 24-item self-report measure for use with parents of children ages 3–12.

**Objective:**

We describe the development and evaluation of the psychometric properties of the BPQ, which was designed to include two subscales: warm and responsive parenting (WRP) and harsh parenting (HP).

**Participants and setting:**

The sample included an equal number of male and female caregivers (*n* = 480) from 240 families in North Lebanon that took part in a randomized controlled trial (RCT) of the Caregiver Support Intervention. Study participants were primarily Syrian (95.2%); others were Lebanese (4.2%) and Palestinian (0.6%).

**Methods:**

The development of the BPQ is described. To assess its psychometric properties, we used data from the RCT for which the instrument was developed. Confirmatory factor analysis (CFA) was used to examine the factor structure of the BPQ. The reliability of the BPQ and its potential subscales was assessed by examining the internal consistency using Cronbach’s alpha and McDonald’s omega. Multi-group CFA was conducted to identify if the same two constructs were being measured across mothers and fathers.

**Results:**

A two-factor model best fit the data, with 16 items loading on WRP and seven items on HP, corresponding to the two parenting dimensions the scale was designed to assess. Internal consistency was good: full scale ɑ=0.83, WRP ɑ=0.86, and HP ɑ=0.76. One item not loading on either subscale was retained for theoretical reasons.

**Conclusions:**

The BPQ is a brief culturally grounded self-report measure of parenting validated for use with Middle Eastern Arabic speaking parents. The data for this paper come from a pre-registered trial: ISRCTN33665023.

**Supplementary Information:**

The online version contains supplementary material available at 10.1186/s40359-025-02395-8.

A growing body of evidence points to the role of the family environment in mediating the impact of armed conflict and forced migration on children’s mental health [1, 2]. Specifically, persistently elevated caregiver stress and distress appear to increase the frequency of harsh parenting and to decrease the use of warm and responsive parenting [3, 4]. Although compromised parenting represents only one pathway by which caregiver stress and distress can impact children’s mental health [5], the available evidence suggests that it is an important mediator of the relationship between caregiver and child wellbeing in settings of adversity [6–8] Recognizing the importance of supporting caregiver wellbeing and strengthening parenting in conflict-affected communities, humanitarian organizations and researchers have partnered to adapt or develop a variety of caregiver-focused interventions [6, 9, 10]. Some focus primarily on strengthening parenting knowledge and skills, while others include a substantive focus on improving caregivers’ own mental health and psychosocial wellbeing [11–14]. Regardless of their specific focus, caregiver interventions generally share the proximal aims of reducing the use of harsh parenting and increasing the frequency of warm and responsive parenting, the two dimensions of parenting most commonly linked to children’s mental health in studies of conflict-affected populations specifically [3] and families living in adversity more generally [8].

***The assessment of parenting***

A key element in the evaluation of caregiver interventions is the psychometrically and culturally sound assessment of parenting. Despite concerns about the limitations of self-report questionnaires due to the risk of response bias [15, 16], they remain the most common method of assessing parenting in low-resource settings due to their minimal cost and ease of administration relative to other methods such as in-home observation. However, most parenting questionnaires have been developed in high-income countries (HICs), while conflict-affected populations reside overwhelmingly in low- and middle-income countries (LMICs). Although several dimensions of parenting have been found to be consistently salient across diverse cultural contexts (e.g., warmth and responsiveness, discipline, control and monitoring), there is considerable cross-cultural variation in the understanding and behavioral manifestation of these dimensions [17].

Consequently, a critical task in the evaluation of caregiver interventions in LMICs is the development or adaptation of parenting measures to ensure that they are both psychometrically sound and culturally appropriate. However, with several notable exceptions [13, 18–21], the validity, cultural fit, and acceptability of parenting measures used in humanitarian settings, and in LMICs more broadly, has often been assumed based on research with other populations, rather than empirically established for the specific population being studied [22]. Although back-translation of questionnaire items has become standard practice, expert panel review and cognitive interviewing—meant to ensure that items are understood as intended, acceptable to ask, and meaningful within the local cultural context— remain underutilized, as reflected in the description of methods in reports of RCTs. Similarly, although researchers often report the internal consistency of the parenting measures used in their studies based on their study sample, validity is often assumed based on a measure’s prior use with culturally different populations and is not reported for the study population. We believe this is an inappropriate assumption, as it overlooks the powerful influence of culture on the understanding and expression of mental health constructs [23]. Hall and his colleagues also caution against assuming good validity based on acceptable reliability. They demonstrated in their research with Somali refugees that questionnaires used in novel populations can demonstrate good internal consistency yet have unacceptably low validity, thus precluding meaningful interpretation of research findings [24].

***Development of the Brief Parenting Questionnaire***

The Brief Parenting Questionnaire (BPQ) was originally developed for use in the evaluation of the Caregiver Support Intervention (CSI), a nine-session group intervention for conflict-affected caregivers of children aged 3–12 [25]. The CSI aims to strengthen parenting through a dual focus on improving caregiver wellbeing and increasing participants’ knowledge and use of positive parenting concepts and methods. In preparation for a pilot RCT of the CSI with Syrian refugees in Lebanon [26], we searched for brief self-report measures of parenting that [1] had been validated for use with Middle Eastern Arabic-speaking parents; [2] could be used with parents of children in the broad age range of 3–12; and [3] assessed parental warmth and responsiveness, as well as harsh parenting, the two dimensions of parenting targeted by the CSI. By harsh parenting, we refer to the behavioral expression of anger in ways that are disproportionate to the misbehavior of a child and are likely to evoke significant fear or other emotional distress by children. By warm and responsive parenting, we refer to nurturing, supportive behaviors likely to evoke feelings of safety and comfort, and demonstrated to foster secure attachment in children [27]. Our emphasis on instrument brevity reflected our plan to administer our assessment battery orally (using a tablet-based software program), due to the variable literacy levels in the study population. After extensive review of existing parenting questionnaires, we were unable to find any measures that met al.l of our criteria. Although we found acceptably short versions of existing measures, they covered too narrow of an age range of index children for our study. Moreover, although several parenting measures have been translated into Arabic, we found none that had been validated in any Middle-Eastern Arabic-speaking population. Finally, there was a consensus within our Lebanon-based research team that the wording of several of the harsh parenting items on existing measures was likely to be perceived as judgmental and provocative in the culturally and religiously conservative Syrian communities we were working with. The team expressed concern that this might discourage honest responding. This concern echoed the caution expressed by Morsbach and Prinz [16] who described the potentially adverse impact on truthful responding of intrusive or otherwise socially undesirable harsh parenting items, in their discussion of self-report parenting measures [16]. Finally, the team also felt that numerous items on existing measures were difficult to translate meaningfully into Arabic.

Although we had not planned on developing a new parenting questionnaire, the lack of an empirically validated brief measure for use with Middle Eastern Arabic-speaking parents, combined with concerns about the cultural fit and translatability of items on existing measures, led to our decision to create a new measure, the Brief Parenting Questionnaire (BPQ).

The aims of this study are:

1. To describe the field testing and validation of the BPQ with Syrian refugees in Lebanon. We begin by briefly describing the initial steps of item development, translation, cognitive interviewing, back-translation, and pilot testing in a sample of 151 primarily Syrian caregivers.
2. To evaluate the psychometric properties of the BPQ, in an RCT sample of 480 caregivers from 240 families, 95% of whom were Syrian refugees, including: (i) scaling assumptions or the extent to which each BPQ item contributes to two sub-scales producing a total parenting score; (ii) reliability or the ability of BPQ to yield consistent estimates of the two constructs under consideration across item responses (internal consistency), and; (iii) assess metric invariance to identify if the same two constructs were being measured across mothers and fathers.

## Methods

### Development of the BPQ

#### Stage 1: item generation

The item generation process was led by a Lebanon-based psychologist bilingual in Arabic and English, with extensive clinical and research experience with Syrian families. Two primary subscales were planned, in keeping with the research and the primary aims of the CSI: warm and responsive parenting (henceforth WRP), and harsh parenting (henceforth HP). Although we recognize that there are other important dimensions of parenting we might have included (e.g., control and monitoring), our decision to focus primarily on WRP and HP reflected the considerations already mentioned, namely, their salience in the literature on parenting in adversity, the need for instrument for brevity, and their centrality as targets of the CSI. After reviewing existing parenting measures that assess these dimensions and carefully considering the aims and content of the CSI, items for the BPQ were developed in English with wording that could readily be translated into formal Arabic, with content and phrasing that were intended to be culturally meaningful and socially acceptable. Guidance and feedback were provided by our Lebanon-based research team and by a group of Amsterdam-based child psychologists and parenting specialists. Items were adapted from existing measures where suitable, and new items were added as needed to ensure coverage of the latent constructs of the full measure and the hypothesized subscales. An initial pool of 24 items was developed that included 14 WRP items and five HP items, with an additional five items putatively related to positive discipline (e.g., use of praise, responding calmly in the face of child misbehavior).

#### Stage 2: initial translation

The measure was then translated into Arabic by two bilingual members of the research team. Care was taken to ensure the ease of understanding, cultural acceptability, and linguistic accuracy of all items in Arabic. Disagreements about the translation of several items were resolved through discussion until agreement was reached on the best translation.

#### Stage 3: cognitive interviewing

We then conducted cognitive interviewing of the translated BPQ with two groups of Syrian women (*n* = 10) and one group of Syrian men (*n* = 10). The aim of the cognitive interviewing was to assess the extent to which each item was understood as intended, made sense culturally, and was perceived as acceptable to ask. Feedback from the cognitive interviews led to minor changes on several items to ensure ease of understanding and cultural fit. All items, including those assessing harsh parenting, were deemed acceptable to ask. We had planned to conduct a second group with men; however, we cancelled it because we had reached saturation with the first three groups; that is, no new information was forthcoming by the time we completed the three cognitive interviewing sessions.

#### Stage 4: back-translation

The BPQ was then back-translated into English by a bilingual research assistant blind to the original English version of the questionnaire. The original and back-translated versions were compared, and minor adjustments were made to the wording of several items on both the Arabic and English versions to further ensure both their comparability and comprehensibility. Beyond that, no additional adjustments were indicated.

#### Stage 5: pilot testing

The BPQ is Likert scaled, with answer choices including *rarely* (1 point), *sometimes* (2 points), or *often* (3 points). Harsh parenting items are reverse scored to yield a total scale score in which higher scores reflect more positive parenting. The answer choices were included in the cognitive interviewing process, and were also translated and back-translated as described above.

The 24 item BPQ was administered, along with the other measures in our assessment battery, in a pilot RCT of the CSI [26]. The sample was comprised of 151 Syrian caregivers, 79 women and 72 men, and included 72 couples. Questionnaire items were read out loud by trained research assistants, and data were gathered using the tablet-based software program Kobo (https://www.kobotoolbox.org/). The internal consistency of the full measure was good (⍺=0.87), as it was for the WRP and HP subscales (⍺=0.84 and ⍺=0.76, respectively). Twenty of the 24 items had item-total correlations above 0.40, three were between 0.20 and 0.39, and one item had an item-total correlation of 0.12. That item, which assessed the ability to match consequences with the severity of a child’s misbehavior, was retained for theoretical reasons despite its weak correlation with the full measure. Although the positive discipline items did not form a reliable subscale, they contributed to the reliability of the full measure, and are theoretically meaningful; consequently, they were retained in the BPQ, but not treated as a subscale in any analyses.

### Methods for the psychometric evaluation of the BPQ in the present study

#### Design

This study used baseline data from a parallel group superiority RCT of the CSI. The protocol for that trial and the results of the study have been published previously [7, 12, 28].

#### Setting and context

The trial was conducted in North Lebanon, an impoverished region that hosts a high number of the Syrian refugees who fled to Lebanon after the start of the Syrian war in 2011. During the study (2019–2020), Lebanon experienced a severe economic crisis and the outbreak of the COVID-19 pandemic. These factors increased the percentage of Syrian refugees living in extreme poverty from 55 to 89% [29].

The sample included an equal number of male and female caregivers (*n* = 480) from 240 families. The majority of the study participants had Syrian nationality (95.2%), the rest of the sample consisting of Lebanese (4.2%) and Palestinians (0.6%). Inclusion criteria were Arabic speaking Syrian refugees or residents from UNOCHA-defined “highly vulnerable” host communities; caring for a child in the range of 3–12 years old; and no recent involvement in a parenting or stress reduction program. Participant demographics can be seen in Table 1.

**Table 1:** Demographic characteristics and summary of parenting measure of caregivers for overall study sample and by gender (*n* = 480)

		Total (*n* = 480)	Females (*n* = 240)	Males (*n* = 240)
Relationship to the child	Mother Father Grandmother Grandfather Other relative	48.5 47.9 0.8 1.3 1.3	97.1 0 1.7 0 0.8	0 95.8 0 2.5 1.7
	Non-relative guardian	0.2	0.4	0
Type home	Apartment House Tented settlement Other	37.7 35.4 1.9 25.0	30.8 41.3 1.7 26.3	44.6 29.6 2.1 23.8
Nationality	Syrian Lebanese Palestinian	95.2 4.2 0.6	95.0 4.6 0.4	95.4 3.8 0.8
Highest education	No schooling Primary Secondary High school Vocational University	6.9 39.2 33.5 11.5 4.2 4.8	6.7 41.7 33.3 13.3 3.3 1.7	7.1 36.7 33.8 9.6 5.0 7.9
Working	Yes No	22.7 77.3	7.1 92.9	38.3 61.7
Parenting total	Mean Median Standard Deviation (SD) (Interquartile range (IQR)	60.2 61 7.1 9	59 60 6.9 8	61.4 63 7.1 9
Harsh Parenting total	Mean Median Standard Deviation (SD) (Interquartile range (IQR)	7.5 7 2.1 3	7.3 7 1.9 3	7.7 7 2.3 3
Warm and Responsive Parenting total	Mean Median Standard Deviation (SD) (Interquartile range (IQR)	36.1 37 4.7 5.5	36.1 37 4.8 6	36.1 37 4.5 6

#### Psychometric evaluation of the BPQ

Descriptive statistics were calculated for all participants. For the BPQ, scores were calculated at both the item and the scale level. The psychometric analysis of the BPQ focused on confirming the scaling assumptions of the BPQ, and evaluating scale and subscale reliabilities.

#### BPQ scaling assumptions

Polychoric correlations were calculated between each item pair to ensure that all items were providing distinct information. Items were then summed to calculate a total score. Corrected item-total correlations < 0.30 were used to indicate unacceptable fit of the items with the BPQ total score [12] (see Supplementary Table 1).

As described earlier, the BPQ was hypothesized to have two dimensions or subscales, warm and responsive parenting and harsh parenting. In order to confirm the dimensionality of the BPQ, we randomly split our sample into two halves. In our first half sample, we conducted a single-level and two-level exploratory factor analysis (EFA). First, we considered it important to investigate how much of the variance in BPQ item scores could be accounted for by families. If there was little variance at the family level, a single-level EFA would be warranted. According to Muthen and colleagues [30, 31], multilevel structure of the data should be modelled when intraclass correlation coefficients (ICCs) are *≥* 0.10 [28]. To calculate ICCs, a random intercept model was run with caregivers at Level 1 and families at Level 2, On the caregiver level, ICCs varied across items between 0.001 and 0.329, with six items being ≥ 0.10 meaning that some items may be influenced at the family level, and we wanted to account for that. This indicated that a two-level EFA was warranted; however, the overall fit indices should be compared with the single-level solution. The factor structure was thus explored by conducting two-level EFAs on the 24 items with combinations of increasing numbers of factors up to 5 on the two levels (the factor structure with more than 5 factors was not tested as factor eigenvalues were below 1). Factors were rotated to the Oblimin criterion. We only wanted to account for the clustering of caregivers within families but were not interested in a factor structure at the family-level. Therefore, we focused on the factor structure of the total correlation matrix compared to the pooled within-correlation matrix to validate the structure of the BPQ. Additionally, we examined the factor structure of the between-correlation matrix and compared it to the total and pooled within-correlation matrices, only to see if there were large substantial differences and use that as input for the confirmatory factor analysis. Due to the small number of response categories (three) and the relatively high skewness and kurtosis of many items (see Supplemental Table S2), the data were analysed as ordered categorical (ordinal), using the robust weighted least squares estimator due to the categorical nature of the response items [30, 31].

Model fit was tested with the use of Confirmatory Factor Analysis using the chi-square test of exact fit, whereas approximate model fit was assessed with the root mean square error of approximation (RMSEA), the comparative fit index (CFI). For RMSEA, a close model fit is considered for values of 0.05 or less, whereas point estimates of 0.05 to 0.08 are considered an acceptable level of approximation [32] with a stringent upper limit of 0.07 suggested by Steiger [33]. Values of 0.10 and above indicate bad fit [33, 34]. CFI values of 0.96 or higher combined with SRMR levels of 0.09 or lower are considered acceptable [30]. Supplementary Table 3 (please see online appendix) displays fit indices for various combinations of numbers of factors derived from the single-level and two-level factor analyses. In general, fit was adequate. The solution we selected was chosen primarily on the grounds of simple structure, screeplot and indicated by the smallest number of items with more than one loading > 0.40 or < − 0.40. Multi-group confirmatory factor analysis (CFA) was conducted to identify if the same two constructs were being measured across mothers and fathers. To evaluate overall model fit, the comparative fit index (CFI) and the root mean square error of approximation (RMSEA) were calculated. A CFI value of greater than 0.90 indicates adequate fit to the data [30]. A value of RMSEA < 0.05 indicates close fit, values between 0.05 and 0.08 suggest adequate model fit, and values > 0.10 suggest poor model fit. The standardized root mean squared residual (SRMR) was also calculated to examine how the model functions to reproduce the relationships from the input covariance matrix, with an SRMR of < 0.1 considered acceptable [35, 36].

#### Reliability

The reliability of the BPQ and its potential subscales was assessed by examining the internal consistency using Cronbach’s α and McDonald’s omega [37], with a criterion of α and omega > 0.70 indicative of adequate internal consistency for the full measure and each subscale. α > 0.90 were also flagged, as this may indicate item redundancy [38, 39].

## Results

Median BPQ total score was 63 (Interquartile range: 57–66) with a possible range of 24–72. Median score for the HP sub-scale was 7 (Interquartile range: 6–9) and for WRP it was 37 (Interquartile range: 34-39.5) The frequency and percentage of response category endorsement for each BPQ item is displayed in Supplementary Table S2.

### BPQ scaling assumptions

Identification of factors with the use of single-level and two-level exploratory and confirmatory factor analysis is shown in Supplemental Table 3. Model fit was acceptable for several of the factor solutions investigated for the single-level, total matrix, pooled within matrix and between matrix solutions. However, considering model fit, eigenvalues, simple structure, and interpretability, two factors on the between-level solution offer the best fit to the data. The first factor is *warm and responsive parenting (WRP)*, and includes 17 items. The second factor is *harsh parenting (HP)* and includes six items. One item did not load on either of the two factors: “When my child misbehaves, I’m able to use consequences that match the severity of the misbehaviour” (see Table 2).

**Table 2:** Factor loadings of BPQ items with identified BPQ latent factors with the use of the two-level exploratory factor analysis

	Parental warmth	Harsh parenting
1. When I see my child, I feel happy	0.5925	0.1258
2. I spend time playing or talking with my child	0.7173	-0.1188
3. I am interested in playing or talking with my child	0.7379	-0.0423
4. I hug and kiss my child	0.7559	-0.1903
5. When my child wants my attention, I tell them not to bother me	-0.1015	0.4788
6. When my child wants my attention, I show a lot of interest	0.6661	-0.0427
7. I do things that make my child feel that I love him/her	0.7227	-0.1078
8. I can make my child feel better if he is sad	0.6196	-0.1511
9. I can make my child feel better if he is afraid	0.5721	-0.069
10. I ask my child what he/she is thinking or feeling	0.6740	0.0396
11. I ask my child about what he/she is doing (e.g., what he/she is playing with/drawing/singing; about his day; about what is happening in school; about his friends, etc.)	0.5238	0.0318
12. I enjoy spending time with my child	0.7159	-0.0052
13. I feel close to my child	0.7917	-0.0909
14. When I interact with my child, it is in a friendly or gentle way	0.7378	-0.3178
15. When I interact with my child, it is in a harsh or angry way (R)	-0.3531	0.6151
16. When my child behaves badly, I respond calmly	0.4362	-0.6042
17. When my child behaves badly, I speak to them harshly or shout at them (R)	-0.4463	0.5282
18. When my child behaves badly, I hit him/her (R)	-0.1839	0.8525
19. I threaten my child in ways that frighten them (R)	-0.1642	0.4106
20. When my child annoys me (talking too much, whining), I hit him/her (R)	-0.3592	0.7661
21. When my child behaves well, I praise him/her	0.4690	-0.4267
22. When my child tries to do something but fails, I praise them for their effort	0.4944	-0.1847
23. When my child does not do what I ask him/her to do, I am able to respond without losing my temper	0.3533	-0.2969
24. When my child misbehaves, I’m able to use consequences that match the severity of the misbehavior.	0.1543*	0.1621*

* Item 24 did not load heavily on either of the two subscales

### Psychometric evaluation of the BPQ and the WRP and HP subscales

The results of the psychometric evaluation of the BPQ are presented in Table 3.

**Table 3:** Summary of psychometric properties of the BPQ

Psychometric property	Constituent parts	*N* = 480
**Scaling assumptions** ^**1**^	Inter-item polychoric correlations (Mean, Range)	Mean = 0.37, Range:=-0.01-0.76
	Polychoric Item-total correlations	0.17 to 0.68
**Reliability**		Internal consistency
	BPQ total	α = 0.86, omega = 0.87
	Warm and Responsive Parenting	α = 0.83, omega = 0.84
	Harsh Parenting	α = 0.76, omega = 0.77
Model fit for single-level CFA	2 factors	Interpretable and acceptable fit SRMR = 0.07 RMSEA = 0.072 (0.063-0.82) TLI = 0.894 CFI = 0.873
	3 factors	Not substantially better fit, not interpretable (too many cross loadings) SRMR = 0.06 RMSEA = 0.057 (0.048–0.069) TLI = 0.855 CFI = 0.853
	4 factors	Not interpretable (too many cross loadings) SRMR = 0.06 RMSEA = 0.048 (0.040–0.062) TLI = 0.864 CFI = 0.910
	5 factors	Not interpretable (too many cross loadings) SRMR = 0.05 RMSEA = 0.041 (0.033–0.055) TLI = 0.937 CFI = 0.963

^1^ See Fig. 2 for results of multi-group confirmatory factor analysis and Table S1 for the polychoric item-total correlations

**Fig. 1:**
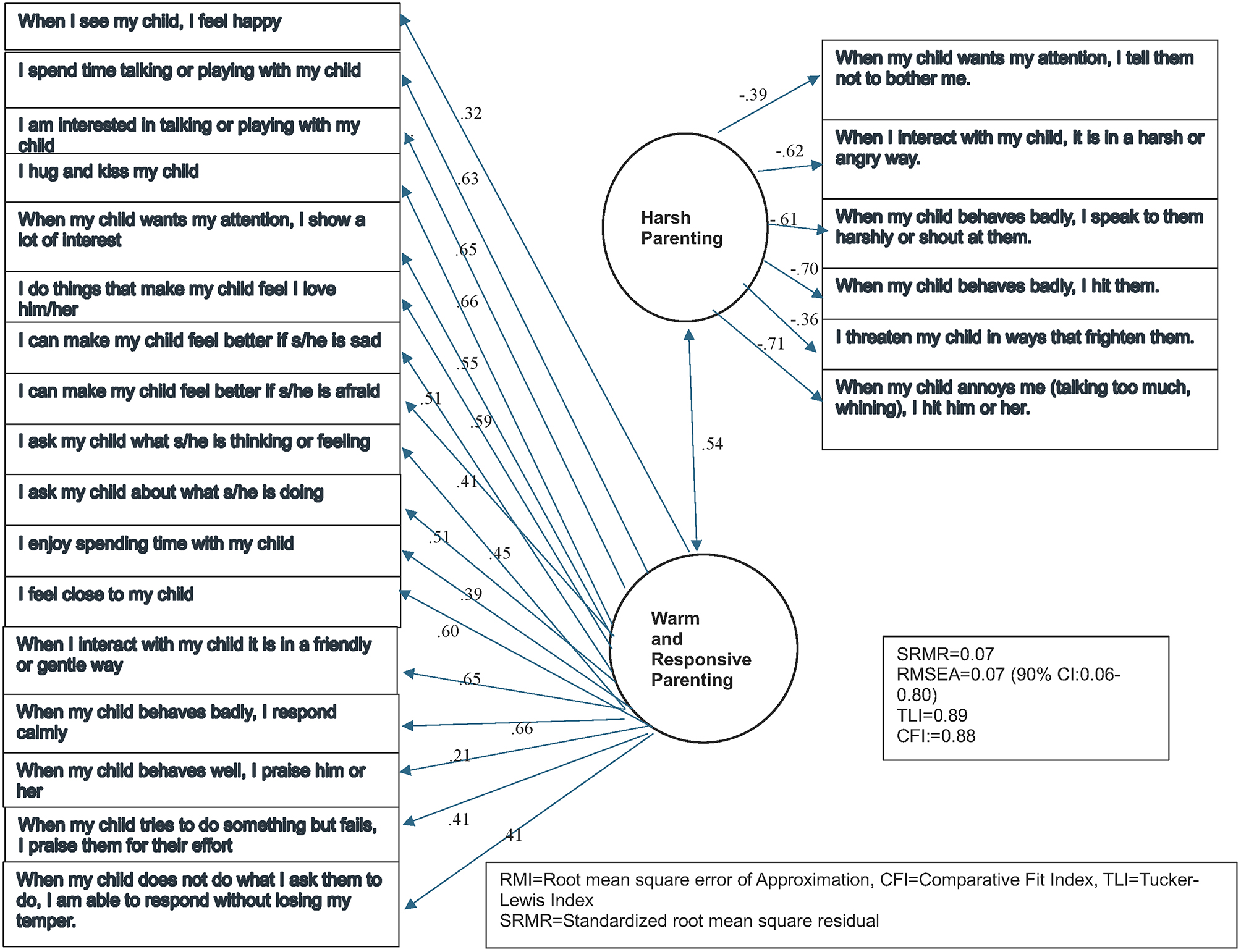
Confirmatory Factor Analysis (CFA) of Brief Parenting Questionnaire scale with items relating to warm and responsive parenting and harsh parenting. Standardised loadings are presented in the graph. RMSEA, Root mean square error of approximation; CFI, Comparative fit index; SRMR, Standardised root mean square residual

### BPQ scaling assumptions

Polychoric correlations did not indicate item-redundancy with an average correlation of 0.37 in the total sample (average range 0.11–0.76). The corrected item-total correlations were generally in the moderate range (0.38–0.68 in total sample) with the exception of one item, “When my child misbehaves, I’m able to use consequences that match the severity of the misbehaviour”. The rest of the 23 BPQ items all loaded significantly on two dimensions with standardized loadings by ranged from 0.21 to 0.66 for the WRP subscale and from − 0.39 to -0.71 for the HP subscale.

**Fig. 2:**
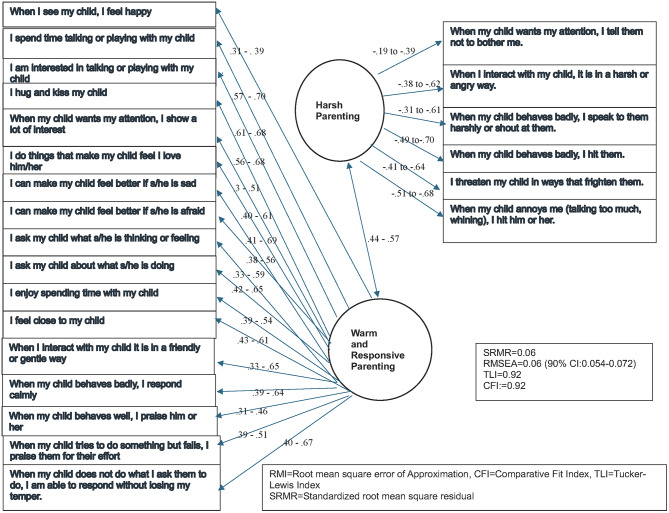
Multi-group CFA model for BPQ by caregiver gender. 1 The ranges for the loadings and errors reflect the standardised figures for male and female caregivers. RMSEA, Root mean square error of approximation; CFI, Comparative fit index; SRMR, Standardised root mean square residual

Significant loadings were seen for each item across males and females. The two-factor CFA solution for BPQ had a reasonable fit, as represented in Fig. 1. The RMSEA value of 0.07, and SMSR < 0.10 suggest acceptable model fit and CFI 0.88 and TLI 0.89.

### Reliability

Cronbach’s alpha and McDonald’s omega coefficient for the 24-item BPS was good at 0.86 and 0.87; for the WRP subscale at 0.83 and 0.84, and was acceptable for HP subscale at 0.76 and 0.77.

## Discussion

The development and validation of the 24-item Brief Parenting Questionnaire (BPQ) addresses the need for brief, culturally grounded, and psychometrically sound parenting measures for use in low-resource settings. Using a rigorous, stepwise methodology similar to that advocated by van Ommeren and colleagues [40], we first sought to ensure the comprehensibility, acceptability, relevance, and translatability of all items with Arabic-speaking Syrians in Lebanon. Although other parenting measures have been used in research with Arabic-speaking Middle Eastern caregivers, there are few published descriptions of the steps taken to ensure the appropriate cultural adaptation of questionnaire content and to assess whether items were understood as intended.

The results of our CFA suggest that a two-factor solution fits the data well, with reliable subscales reflecting warm and responsive parenting (17 items) and harsh parenting (6 items). Although we believe the BPQ total score yields a useful global metric of parenting, the subscales, which showed acceptable (HP) to good (WRP) internal consistency, are particularly useful in that they allow for the assessment of change in the two dimensions of parenting behavior that have been consistently related to child mental health outcomes in families experiencing high levels of caregiver stress and distress [3]. We included items to comprise our subscale based on strong theoretical value. One item “When my child misbehaves, I’m able to use consequences that match the severity of the misbehavior” only seemed to be weakly correlated with the two subscales but the inclusion/exclusion of this item needs to be tested in different populations. The multi-dimensional nature of the scale is also supported by the fairly high and reliable alpha values of the warm and positive parenting subscale (alpha = 0.83; omega = 0.84) and harsh parenting subscale (alpha = 0.76; omega = 0.77). In addition, scree plot (Figure S1) and model fit indices for the one-factor solution were not within an acceptable range (RMSEA = 0.15, CFI = 0.8, SRMR = 0.1, CFI = 0.7 TLI = 0.8) and the model fit was considerably improved with the two-factor solution (RMSEA = 0.07, CFI = 0.8, SRMR = 0.07, CFI = 0.8 TLI = 0.8). At the end, a decision was made that was based on the best fit indices, as well as on the interpretability of the two-factor solution which was aligned with the theoretical rationale and framework for the development of the BPQ. This makes it a potentially useful instrument for assessing the impact of parenting interventions on these key dimensions. This may be particularly useful when an intervention primarily aims to reduce harsh parenting, for example, or when an absence of significant change on the BPQ total score masks meaningful improvement on either the WRP or HP subscales.

An additional benefit of the BPQ is its applicability for use with parents of children ages 3–12, a comparatively large age range relative to many other parenting measures. In a subsequent study, we hope to assess the utility of the BPQ with caregivers of early teens. The age range in this validation study reflects the focus of the RCT from which the data were drawn; the Caregiver Support Intervention is specifically designed for parents of children ages 3–12. For children under the age of three, several cross-culturally validated measures already exist [35].

### Limitations & future directions

The BPQ is meant to assess change over time in parenting behavior. This suggests the importance of assessing its test-retest reliability, so that evaluators can be confident that changes on the measure reflect actual changes in parenting and not test-retest variability inherent in the questionnaire. Although we did not do so in the current study, we plan to assess the test-retest reliability of the BPQ in a separate study.

There are several types of validity that can be assessed when examining the psychometric properties of newly developed or adapted measures. In the present study, we used CFA to examine the factor structure of the BPQ, a widely used approach to assessing construct validity. In a subsequent study, we hope to assess the convergent validity of the BPQ by examining its correlation with another validated parenting measure. The challenge for doing so lies in the lack of validated parenting measures for use in Middle Eastern Arabic populations—a key reason that we developed the BPQ.

The BPQ only assesses caregivers’ own ratings of their parenting. To address this limitation, we are currently planning the development of a child-reported version of the BPQ that can be completed by school-age children. We also note that the items on the harsh parenting subscale of the BPQ do not include all.

possible harsh parenting behaviors, such as being emotionally cold to a child or ignoring them. Invariably, any decision to prioritize instrument brevity comes to some extent at the cost of comprehensiveness. However, we note that even with just six items, the HP subscale demonstrated good internal consistency.

Although we developed the BPQ for use in an RCT of a psychosocial intervention with Arabic-speaking Middle Eastern families, we believe the measure should work well in other populations as well, since the subscales in the BPQ reflect transculturally salient dimensions of parenting. However, future research will need to explore what sort of cultural and linguistic adaptations might be needed prior to using the BPQ in other cultural contexts. Moreover, the construct validity found in this study cannot be assumed to generalize across different populations. We are currently working on the adaptation of the BPQ for use in South Sudan [36].

Finally, we note the high mean BPQ scores among both female and male participants in this study. Although the standard deviations suggest considerable variance in total and subscale scores, the high means do raise the possibility of a social desirability bias that may have influenced participant responses. This is an inherent drawback in any self-report parenting measure, particularly when administered orally by research assistants, as was done in this study. This underscores the value of gathering data on parenting using multiple methods and from multiple sources, such as the child-report version we are currently working on.

The 24-item BPQ is a brief and easily administered, reliable, two-dimensional measure of parenting validated for use with Arabic-speaking Middle Eastern caregivers of children ages 3–12. Based on our study of Syrian parents displaced in Lebanon, the BPQ demonstrated good construct validity and internal consistency. Because the measure offers a quick and freely available self-report tool for assessing parenting behavior, it may be particularly valuable in resource-limited settings such as humanitarian crises and LMICs more generally.

## Electronic supplementary material

Below is the link to the electronic supplementary material.

Supplementary Material 1

## Data Availability

The datasets used and/or analyzed during the current study are available from the corresponding author on reasonable request.
