# Supplementary material for: Development and psychometric evaluation of the Brief Parenting Questionnaire
Source: BMC Psychol. 2025 Jan 29;13:85. doi: 10.1186/s40359-025-02395-8 (PMC11780980; doi:10.1186/s40359-025-02395-8)
Supplement: Supplementary file 1 — Supplementary Material 1 [file 40359_2025_2395_MOESM1_ESM.docx]

**Brief Parenting Questionnaire: Supplemental Tables and Figure**

**Table S1.** Polychoric correlations between each item and BPQ total score

| 1.       When I see my child, I feel happy | 0.45 |
| --- | --- |
| 2.       I spend time playing or talking with my child | 0.68 |
| 3.      I am interested to play or talk with my child | 0.67 |
| 4.       I hug and kiss my child | 0.71 |
| 5.       When my child wants my attention, I tell them not to bother me | -0.37 |
| 6.       When my child wants my attention, I show a lot of interest | 0.59 |
| 7.       I do things that make my child feel that I love him/her | 0.64 |
| 8.       I can make my child feel better if he is sad | 0.61 |
| 9.       I can make my child feel better if he is afraid | 0.51 |
| 10.   I ask my child what he/she is thinking or feeling | 0.62 |
| 11.   I ask my child about what he/she is doing (e.g. what he/she is playing with/drawing/singing; about his day; about what is happening in school; about his friends, etc.) | 0.48 |
| 12.   I enjoy spending time with my child | 0.63 |
| 13.   I feel close to my child | 0.67 |
| 14.   When I interact with my child, it is in a friendly or gentle way | 0.73 |
| 15.   When I interact with my child, it is in a harsh or angry way (R) | -0.57 |
| 16.   When my child behaves badly, I respond calmly | 0.66 |
| 17.   When my child behaves badly, I speak to them harshly or shout at them (R) | -0.66 |
| 18.   When my child behaves badly, I hit him/her (R) | -0.54 |
| 19.   I threaten my child in ways that frighten them (R) | -0.38 |
| 20.   When my child annoys me (talking too much, whining), I hit him/her (R) | -0.61 |
| 21.   When my child behaves well, I praise him/her | 0.57 |
| 22.   When my child tries to do something but fails, I praise them for their effort | 0.54 |
| 23.   When my child does not do what I ask him/her to do, I am able to respond without losing my temper | 0.48 |
| 24.   When my child misbehaves, I’m able to use consequences that match the severity of the misbehaviour. | 0.17 |

**Table S2.** Item frequencies and percentages

|  | n(%) | n(%) | n(%) |
| --- | --- | --- | --- |
| 1.       When I see my child, I feel happy | 5(2.08) | 22(9.17) | 213(88.7) |
| 2.       I spend time playing or talking with my child | 37(15.4) | 75(31.2) | 128(53.3) |
| 3.      I am interested to play or talk with my child | 41(17.0) | 62(25.8) | 137(57.0) |
| 4.       I hug and kiss my child | 19(22.5) | 54(22.5) | 167(69.5) |
| 5.       When my child wants my attention, I tell them not to bother me | 71(29.5) | 123(51.2) | 46(19.1) |
| 6.       When my child wants my attention, I show a lot of interest | 16(6.6) | 63(26.2) | 161(67.0) |
| 7.       I do things that make my child feel that I love him/her | 13(5.4) | 50(20.8) | 177(73.7) |
| 8.       I can make my child feel better if he is sad | 25(10.4) | 61(25.4) | 154(64.1) |
| 9.       I can make my child feel better if he is afraid | 18(7.5) | 46(19.1) | 176(73.3) |
| 10.   I ask my child what he/she is thinking or feeling | 48(20.0) | 96(40.0) | 96(40.0) |
| 11.   I ask my child about what he/she is doing (e.g. what he/she is playing with/ drawing/ singing; about his day; about what is happening in school; about his friends, etc.) | 17(7.0) | 71(29.5) | 152(63.3) |
| 12.   I enjoy spending time with my child | 22(9.1) | 50(20.8) | 168(70.0) |
| 13.   I feel close to my child | 11(4.5) | 38(15.8) | 191(79.5) |
| 14.   When I interact with my child, it is in a friendly or gentle way | 7(2.9) | 54(22.5) | 179(74.5) |
| 15.   When I interact with my child, it is in a harsh or angry way (R) | 122(50.8) | 92(38.3) | 26(10.8) |
| 16.   When my child behaves badly, I respond calmly | 41(17.0) | 122(50.8) | 77(32.0) |
| 17.   When my child behaves badly, I speak to them harshly or shout at them (R) | 88(36.6) | 108(45.0) | 44(18.3) |
| 18.   When my child behaves badly, I hit him/her (R) | 160(66.7) | 59(24.5) | 21(8.7) |
| 19.   I threaten my child in ways that frighten them (R) | 137(57.0) | 75(31.2) | 28(11.6) |
| 20.   When my child annoys me (talking too much, whining), I hit him/her (R) | 174(72.5) | 51(21.2) | 15(6.2) |
| 21.   When my child behaves well, I praise him/her | 13(5.4) | 30(12.5) | 197(82.0) |
| 22.   When my child tries to do something but fails, I praise them for their effort | 20(8.33) | 82(34.1) | 138(57.5) |
| 23.   When my child does not do what I ask him/her to do, I am able to respond without losing my temper | 32(13.3) | 116(48.3) | 92(38.3) |
| 24.   When my child misbehaves, I’m able to use consequences that match the severity of the misbehaviour. | 72(30.0) | 107(44.5) | 61(25.4) |

**Table S3**. Fit indices for various combinations of numbers of factors derived from a two-level factor analyses.

|  | SRMR | RMSEA (90% CI) | TLI | CFI |
| --- | --- | --- | --- | --- |
| Single-level CFA |  |  |  |  |
| 2 factors | 0.07 | 0.072 (0.063-0.80) | 0.894 | 0.873 |
| 3 factors | 0.06 | 0.057 (0.048-0.069) | 0.855 | 0.853 |
| 4 factors | 0.06 | 0.048 (0.040-0.062) | 0.864 | 0.910 |
| 5 factors | 0.05 | 0.041 (0.033-0.055) | 0.937 | 0.963 |
| Total matrix |  |  |  |  |
| 2 factors | 0.06 | 0.064 (0.056-0.073) | 0.813 | 0.876 |
| 3 factors | 0.05 | 0.054 (0.045-0.064) | 0.865 | 0.903 |
| 4 factors | 0.04 | 0.048 (0.037-0.059) | 0.894 | 0.930 |
| 5 factors | 0.04 | 0.037 (0.023-0.05) | 0.937 | 0.963 |
| Pooled within matrix: |  |  |  |  |
| 2 factors | 0.07 | 0.067 (0.054-0.082) | 0.766 | 0.809 |
| 3 factors | 0.06 | 0.059 (0.043-0.074) | 0.821 | 0.869 |
| 4 factors | 0.05 | 0.051 (0.033-0.069) | 0.861 | 0.909 |
| 5 factors | 0.05 | 0.044 (0.02-0.064) | 0.894 | 0.939 |
| Between matrix: |  |  |  |  |
| 2 factors | 0.07 | 0.06 (0.046-0.075) | 0.833 | 0.863 |
| 3 factors | 0.06 | 0.048 (0.029-0.065) | 0.892 | 0.921 |
| 4 factors | 0.05 | 0.037 (0-0.057) | 0.934 | 0.957 |
| 5 factors | 0.04 | 0.012 (0-0.045) | 0.99 | 0.994 |

*SRMR >.10=poor, .06-.08 =acceptable, <.06=excellent; TLI >.90 =good; CFI < .90=poor, >.90=acceptable, >.95=excellent*


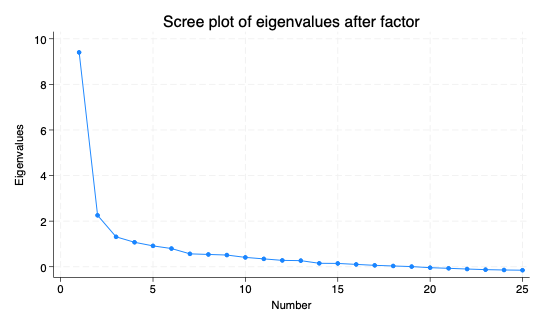


Figure S1. Scree plot of Exploratory Factor Analysis
